# Factors associated with care- and health-related quality of life of caregivers of children with juvenile idiopathic arthritis

**DOI:** 10.1186/s12969-022-00713-7

**Published:** 2022-07-23

**Authors:** Luiza R. Grazziotin, Gillian Currie, Marinka Twilt, Maarten J. IJzerman, Michelle M. A. Kip, Hendrik Koffijberg, Gouke Bonsel, Susanne M. Benseler, Joost F. Swart, Sebastiaan J. Vastert, Nico M. Wulffraat, Rae S. M. Yeung, Wineke Armbrust, J. Merlijn van den Berg, Deborah A. Marshall

**Affiliations:** 1 Department of Community Health Sciences, Cumming School of Medicine, University of Calgary, Room 3C56, Health Research Innovation Centre, 3280 Hospital Drive NW, Calgary, AB T2N 4Z6 Canada; 2 McCaig Institute for Bone and Joint Health, University of Calgary, Room 3C56, Health Research Innovation Centre, 3280 Hospital Drive NW, Calgary, AB T2N 4Z6 Canada; 3 O’Brien Institute for Public Health, University of Calgary, Room 3C56, Health Research Innovation Centre, 3280 Hospital Drive NW, Calgary, AB T2N 4Z6 Canada; 4 Alberta Children’s Hospital Research Institute, University of Calgary, Room 3C56, Health Research Innovation Centre, 3280 Hospital Drive NW, Calgary, AB T2N 4Z6 Canada; 5 Department of Paediatrics, Cumming School of Medicine, University of Calgary, Calgary, Alberta Canada; 6 Section of Rheumatology, Department of Paediatrics, Cumming School of Medicine, University of Calgary, Calgary, Alberta Canada; 7 Department of Health Technology and Services Research, Faculty of Behavioural, Management and Social Sciences, Technical Medical Centre, University of Twente, Enschede, Netherlands; 8 EuroQol Research Foundation, Rotterdam, the Netherlands; 9 Alberta Health Services, Calgary, Alberta Canada; 10 Department of Pediatric Immunology and Rheumatology, Wilhelmina Children’s Hospital / UMC Utrecht, Utrecht, Netherlands; 11 Faculty of Medicine, Utrecht University, Utrecht, Netherlands; 12 Departments of Paediatrics, Immunology and Medical Science, The Hospital for Sick Children, University of Toronto, Toronto, Canada; 13 Wineke Armbrust University of Groningen, University Medical Center Groningen (UMCG), Beatrix Childrens Hospital, Dept Pediatric Rheumatology-Immunology, Groningen, Netherlands; 14 Department of Pediatric Immunology, Rheumatology and Infectious Diseases, Emma Children’s Hospital, Amsterdam University Medical Centers (Amsterdam UMC), University of Amsterdam, Amsterdam, Netherlands

## Abstract

**Objective:**

This study investigates the relationship of child, caregiver, and caring context measurements with the care-related quality of life (CRQoL) and health-related quality of life (HRQoL) of caregivers of children with juvenile idiopathic arthritis (JIA).

**Methods:**

We performed a cross-sectional analysis of baseline data on caregivers of children with JIA from Canada and the Netherlands collected for the “Canada-Netherlands Personalized Medicine Network in Childhood Arthritis and Rheumatic Diseases” study from June 2019 to September 2021. We used the CRQoL questionnaire (CarerQoL), adult EQ-5D-5L, and proxy-reported Youth 5-Level version of EuroQoL (EQ-5D-5L-Y) to assess caregiver CRQoL, caregiver HRQoL, and child HRQoL, respectively. We used a multivariate analysis to assess the relationship between both caregiver CRQoL and HRQoL and patient, caregiver, and caring context measurements.

**Results:**

A total of 250 caregivers were included in this study. Most of the caregivers were from the Netherlands (*n* = 178, 71%) and 77% were females (*n* = 193). The mean CarerQoL scores was 82.7 (standard deviation (SD) 11.4) and the mean EQ-5D-5L utility score was 0.87 (SD 0.16). Child HRQoL and employment had a positive relationship with both caregiver CarerQoL and EQ-5D-5L utility scores (*p* < 0.05), while receiving paid or unpaid help had a negative relationship with both scores (*p* < 0.05).

**Conclusion:**

Our findings indicated that to understand the impact of JIA on families, we need to consider socio-economic factors, such as employment and support to carry caregiving tasks, in addition to child HRQoL.

## Key findings

- This study assessed the CRQoL and HRQoL of caregivers of children with JIA and explored factors associated with these two variables.
- Caregiver CRQoL and HRQoL were both positively associated with child HRQoL and employment status, and negatively associated with receiving paid or unpaid help.
- To understand the impact of JIA on families, we need to consider not only children’s disease activity status, but also socio-economic factors affecting caregivers.

## Introduction

*‘Arthritis is a family disease’* exemplifies the experience of parents living with and caring for a child with juvenile idiopathic arthritis (JIA) [1]. JIA is an umbrella term for a group of rheumatic diseases associated with significant short- and long-term issues, including the risk of functional impairment due to joint swelling, pain and stiffness, growth abnormalities, osteoporosis, and psychological distress [2, 3]. All these problems can impact the health-related quality of life (HRQoL) of children with JIA and their families and are associated with increased morbidity [3–6]. Pharmacological treatment for JIA is pivotal for controlling symptoms and preventing long-term disability [7]. JIA treatment generally improves the child’s health, but it also can cause adverse reactions and creates discomfort with frequent use of needles [8, 9].

Both the disease and its treatment significantly impact the quality of life and work productivity of the caregivers, who are most often the parents [10, 11]. Qualitative research has identified that caregivers face many challenges that affect their well-being, including balancing their child’s demands with their own psychological needs when feeling depressed or stressed, and accompanying the child to the frequent health appointments [12].

Current guidance for economic evaluations recommends the inclusion of both patients and family members’ costs and benefits when assessing cost-effectiveness of health interventions or technologies when using a societal perspective as a scenario analysis [13–15]. One measure of benefits in economic evaluations is generic HRQoL, usually measured using instruments such as EQ-5D, which when linked to ‘value sets’ generate health utility scores, an index measure which reflects values of patients for distinct health states. However, typically, when effects on caregivers are included in pediatric economic evaluations, the focus is restricted to productivity loss as a result of caring (opportunity costs) and out-of-pockets costs [16]. The impact on caregiver’s health and well-being in health utility terms, which can be used to inform economic evaluations and subsequently decision making, has been rarely reported. We identified only one study reporting health utility scores of caregivers of children with JIA, which had a very small sample size (*n* = 47), with less than 17 participants per country [17].

Capturing the impact of JIA on the caregiver should go beyond measuring HRQoL alone, as there is a wide spectre of positive and negative effects [18]. Despite all challenges, caregivers also report positive outcomes on the family level, including closer relationships and a positive readjustment of family priorities [19]. Caregiver’s well-being in JIA appears affected by the child’s overall well-being as expected, but also by the inability to control the child’s pain or fatigue and the provision of care that inflict pain, such as administration of medication at home [19].

Care-related quality of life (CRQoL) instruments have been developed to capture distinct aspects of caring, such as the Adult Social Care Outcomes Toolkit for Carers, Carer Experience Scale, Care-Related Quality of Life (CarerQoL) [20, 21]. Measures like CarerQoL permit analysis of the source of positive and negative impacts and to calculate caregiver-focused utility equivalent scores [20]. A recent study including caregivers of adult patients with dementia, stroke, mental illness, and rheumatoid arthritis revealed that CarerQoL scores were associated with caring context variables, such as the nature of employment, the volume of support and care per week, and the need to provide personal care [22].

So far CarerQoL has been used to measure CRQoL in caregivers of children with autism spectrum, Beta-Thalassemia Major, craniofacial malformations, cystic fibrosis, drug-resistant epilepsy, and neuromuscular disorder [23–27]. To the best of our knowledge, there are no studies reporting CRQoL of caregivers of children with JIA, and on the relation between the health of caregivers and that of the child cared for.

The main aim of this study is therefore to assess the CRQoL and HRQoL of caregivers of children with JIA in Canada and in the Netherlands, and to explore the presence and direction of relationships between health of caregivers (caregiver CRQoL and HRQoL) and child HRQoL and other caring context variables.

## Methods

This study is a cross-sectional analysis of data collected as part of the “Canada-Netherlands Personalized Medicine Network in Childhood Arthritis and Rheumatic Diseases (UCAN CANDU)” between June 2019 to September 2021. The UCAN CANDU is an on-going prospective, multicentre study including all pediatric rheumatology clinics in Canada and the Netherlands which focused on personalized care strategies in JIA through biological monitoring systems. There are three groups of children included in the study: children with a new diagnosis of JIA as per the International League of Associations for Rheumatology (ILAR) classification criteria or children who are starting or discontinuing a biological therapy. Parents and/or caregivers of children younger than 18 years old attending one of the sites were invited to participate. If both parents were present during enrollment, they were asked to select among themselves a person responsible for completion of the questionnaires. We obtained informed consent from all individual parents/caregivers. Ethics approval was granted by the Conjoint Health Research Ethics Board at the University of Calgary (REB17–1563) for Canada and by the Ethical Board of Utrecht (18–474) for the Netherlands.

At baseline, an electronic case report form containing children’s clinical information was completed by a pediatric rheumatologist or a research coordinator. In addition, caregivers were asked to complete a package of questionnaires which includes: 1) report on CRQoL using CarerQoL, 2) report on their own health using adult 5-level version of EuroQoL (EQ-5D-5L), 3) report on child’s health proxy-reported youth 5-level version of EuroQoL (EQ-5D-5L-Y), and 4) a survey to capture additional caregivers’ and caring context characteristics. The questionnaire package was available electronically using an e-Health platform or as paper copy, which were entered electronically by a study team member.

To generate the analytic dataset for this paper, we included caregivers who completed all three CRQoL and HRQoL questionnaires within 30 days of the date of the case report form baseline assessment. Patients and parents included in this paper were enrolled from the following pediatrics sites across Canada and the Netherlands: Alberta Children’s Hospital, British Columbia Children’s Hospital, Children’s Hospital of Eastern Ontario, Children’s Hospital at London Health Sciences Centre, the Hospital for Sick Children Research Institute, IWK Health Centre, Jim Pattison Children’s Hospital, and Montreal Children’s Hospital, Beatrix Children’s Hospital, Emma Children’s Hospital, and Wilhelmina Children’s Hospital.

We treated the data from the Netherlands and Canada as equivalent for both the CRQoL and HRQoL instruments. Therefore, we interpreted any differences in the estimates between the two counties as true differences.

### Clinical data

The clinical data contained information regarding patient’s country, age, sex, time of diagnosis in relation to baseline visit, number of active joints, disease status (i.e., classified by clinicians as active or inactive disease), JIA classification, and treatment information such as ongoing therapy with disease-modifying antirheumatic drugs (DMARDs) or biologics, including the administration mode of current therapy (i.e., oral, subcutaneous, intravenous), during the baseline clinical assessment.

We collected additional information regarding caregiver’s characteristics (i.e., age, sex, education level, and employment status), and caring context (i.e., if caregivers live with their spouse/partner, and level of support from a paid housekeeper or nanny, or unpaid support from family and friends) using a survey.

### Care-related quality of life of caregivers

The CarerQol is a validated instrument which measured CRQoL and consists of a descriptive system (CarerQol-7D) and a visual scale analogue (VAS), CarerQoL-VAS [18]. The CarerQol-7D contains seven domains of caregiving burden. Five of these domains report the potentially negative aspects of caring: relational problems with the care recipient, mental health problems, problems with daily activities, financial problems, and physical health problems. Two domains report on positive experiences from caring: fulfillment, and support. The CarerQol-7D uses three ordinal response categories: no, some, and a lot. The CarerQol-VAS measures happiness with defined endpoints of (0) ‘completely unhappy’ and (10) ‘completely happy’.

The CarerQol-7D descriptive system can be linked to value sets, which generate caregiver-focused utility equivalent scores, an index measure which reflects general population preference values for each one of the 2187 (3^7^) unique care situations. The CarerQoL utility values range from 0 to 100, where 0 represents lowest possible CRQoL and 100 full CRQoL These caregiving states were valued using previously collected preferences from the general public on these states derived from a discrete choice experiment [20, 28]. In this study we used the value set from the Netherlands, since Canadian value sets were not available at the time of this analysis [28].

### Health-related quality of life of caregivers and child

The self-reported version of EQ-5D-5L was used to assess caregiver HRQoL. The EQ-5D-5L is a generic health utility instrument developed by the EuroQol Group [29]. EQ-5D-5L is comprised of two components, a descriptive system, and EQ-5D-5L VAS [30]. The EQ-5D-5L descriptive system consists of five domains (mobility, self-care, usual activities, pain/discomfort, and anxiety/depression) each with five levels (no, slight, moderate, severe, and extreme problems). The EQ-5D-5L VAS records the rated health with defined endpoints of (0) ‘the worst health you can imagine’ and (100) ‘the best health you can imagine’. The proxy-reported version of the preliminary EQ-5D-5L-Y was used to assess child HRQoL [31, 32].

The EQ-5D-5L descriptive system can be linked to value sets, which generate utility scores, an index measure which reflects general population preference values for each one of the 3125 (5^5^) distinct health states. EQ-5D-5L utility values range from < 0 (where 0 is the value of a health state equivalent to dead; negative values representing values worse than dead) to 1 (the value of full health), with higher scores indicating higher utility. For descriptive purposes, the EQ-5D-5L utility scores were calculated using adult Dutch and Canadian value sets depending on the country of residency of the participants [33, 34]. For the regression analysis, given the limited sample size to analyze participants from each country separately, we used value sets from the Netherlands for the whole cohort.

### Statistical analysis

A descriptive analysis of demographic and socioeconomic variables was conducted using frequency measures. The results of the caregiver’s CarerQol, EQ-5D-5L, child proxy-reported EQ-5D-5L-Y questionnaires were reported as proportion of answers for each domain. For the description of CarerQoL and EQ-5D-5L utility scores and VAS, the data were stratified by participant’s country of origin and reported as mean, median, standard deviation, and interquartile range.

We used the Spearman rank test to assess the association among the proxy-reported EQ-5D-5L-Y domains and EQ-5D-5L-Y VAS with both the EQ-5D-5L and CarerQoL domains, utility scores and respective VAS. Spearman’s correlation coefficients were classified as perfect (1), very strong (0.8–0.99), moderate (0.6–0.79), fair (0.3–0.59), poor (0.1–0.29), and none (0–0.09, [35]. Due to multiple tests, we used Bonferroni adjustment and defined a *p*-value lower than 0.05 for statistically significant associations.

We used multivariate regression analysis to explore the relationship between CarerQoL and EQ-5D-5L utility scores and patient characteristics (i.e., age, sex, and disease status), patient’s treatment characteristics (i.e., treatment with medications administered subcutaneously), caregiver’s characteristics (i.e., age, sex, and employment status), and caring situation (i.e., whether caregivers lived with a partner, and whether they received paid or unpaid support with their care-tasks). These variables were selected based on evidence about factors associated with caregiver’s quality of life from the literature [19, 22]. For caregiver’s CarerQoL utility scores analysis, we used a multivariate OLS coupled with robust standard errors to correct for heteroskedasticity [36]. For the analysis of caregiver’s EQ-5D-5L utility scores, we performed multivariable regression using a two-part model to deal with the upward-skewed distribution of the outcome (‘ceiling effect’) [37]. In the first step, we assessed the probability of reaching full health (utility score equals to 1) using a logistic regression. In the second step, we used ordinary least square regression (OLS) for utility scores below 1. Since we used value sets from the Netherlands for the entire sample to perform this analysis, we also included country of origin as an independent variable. We evaluated multicollinearity of independent variables, normality (Shapiro-Wilk test), and homoscedasticity (Breusch-Pagan test). All analyses were performed in R.

## Results

A total of 250 caregivers completed CarerQoL, EQ-5D-5L regarding their own health, and proxy-reported EQ-5D-5L-Y questionnaire regarding their children’s health at baseline. No significant differences were identified between the study sample of 250 participants who completed CarerQoL, EQ-5D-5L, and EQ-5D-5L-Y questionnaires and those who did not fully complete all three questionnaires (*n* = 330), regarding children’s age (*p* = 0.95), sex (*p* = 0.54), joint count (*p* = 0.49), and disease status (*p* = 0.19). However, the proportion of participants with questionnaires completed is higher in the Netherlands (50%) than Canada (32%) (*p* < 0.05).

All caregivers described a parental relationship with the child enrolled in the study. Most caregivers were female (77%, *n* = 193), with a median age of 42 years (IQR 37–46). Most children with JIA were classified as having an active disease (75%, *n* = 187) at baseline. Other characteristics are described in Table 1.

**Table 1:** Baseline characteristics of patients and caregivers included in this analysis

	Patients` characteristics (*n =* 250)	Caregivers’ characteristics (*n =* 250)
Age at baseline, median (IQR), years	12 (8–14)	42 (37–46)
Female, n (%)	155 (62%)	193 (77%)
Country, n (%)		
Canada	72 (29%)	–
Netherlands	178 (71%)	–
JIA classification, n (%)		
Polyarticular JIA RF negative	56 (22%)	–
Polyarticular JIA RF positive	12 (5%)	–
Extended Oligoarticular JIA	20 (8%)	–
Persistent Oligoarticular JIA	28 (11%)	–
Oligoarticular JIA (not classified yet: < 6 months)	44 (18%)	–
Enthesitis-related arthritis	34 (14%)	–
Systemic JIA	16 (6%)	–
Other subtypes	11 (4%)	–
Missing	﻿29 (12%)	–
Duration of disease at baseline, n (%)		
Diagnosis at the baseline visit or after	41 (16%)	–
Up to 12 months before baseline visit	58 (23%)	–
More than 12 months before baseline visit	109 (43%)	–
Missing	45 (18%)	–
Disease status, n (%)		
Active	187 (75%)	–
Inactive	51 (20%)	–
Missing	12 (5%)	–
Active joint count		
Median (IQR)	2 (0–4)	–
Missing, n (%)	11 (4%)	–
Treatment, n (%)		
DMARDs	76 (30%)	–
Biologicals	62 (25%)	–
Subcutaneous DMARDs or biologics	58 (23%)	–
Education, n (%)		
University	–	117 (47%)
College	–	12 (5%)
﻿Technical/Trade school	–	72 (29%)
Grade school	–	4 (2%)
High school	–	25 (10%)
Missing	–	19 (8%)
Employment, n (%)		
Yes	–	192 (77%)
No	–	48 (19%)
Missing	–	10 (4%)
Caregiver lives with spouse/partner, n (%)		
Yes	–	211 (84%)
No	–	22 (9%)
Missing	–	17 (7%)
Extra (paid) help (e.g., house-cleaner, baby-sitter), n (%)		
Yes	–	19 (7%)
No	–	214 (85%)
Missing	–	17 (7%)
Extra (unpaid) help from family, friends, or neighbours, n (%)		
Yes	–	39 (16%)
No	–	194 (77%)
Missing	–	17 (7%)
Adequacy of help at home, n (%)		
Have enough help	–	166 (66%)
Could use more help sometimes/often	–	34 (14%)
Do not have enough help	–	32 (13%)
Missing	–	17 (7%)

*JIA* Juvenile idiopathic arthritis, *IQR* Interquartile range, *RF* Rheumatoid factor, *DMARDs* Disease modifying anti-rheumatic drugs

No missing data was observed within questions from CarerQoL, EQ-5D-5L, or proxy-reported EQ-5D-5L-Y questionnaires. There was less than 8% missing data in patient’s and caregiver’s characteristics, with exception of JIA classification (12%) and date of diagnosis (18%).

### Care-related quality of life of caregivers

**Fig. 1:**
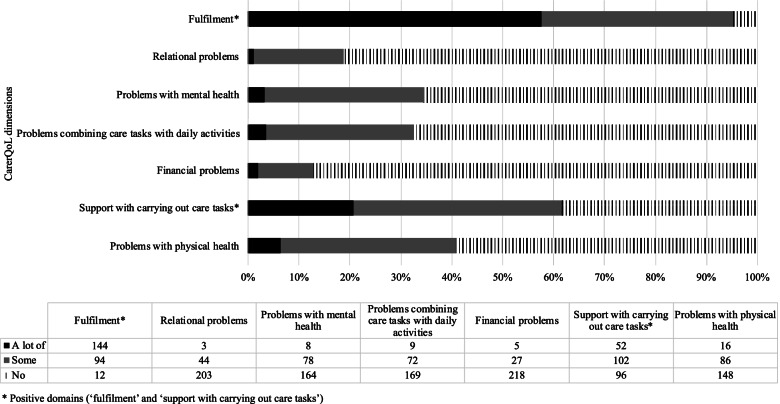
Description of CarerQol in caregivers of JIA children (*n =* 250, including *n =* 72 from Canada and *n =* 178 from the Netherlands)

Figure 1 presents the results on the CarerQol (*n* = 250) for the seven domains separately. Among the negative domains, the ones with higher proportion ‘lot of’ or ‘some’ problems were physical health (39.2%, *n* = 98) and mental health (34.4%, *n* = 86). Of the positive domains, 95.2% (*n* = 238) report ‘a lot of’ or ‘some’ fulfilment from carrying out care tasks. While 61.6% (*n* = 154) of caregivers report at least some support with carrying out care tasks when needed (e.g., from family, friends, neighbours, acquaintances), 38.4% (*n* = 96) reported ‘no’ support. The mean CarerQoL utility score was 80.1 (SD 13.0, IQR 74–88) and 83.7 (SD 10.6, IQR 81–92), for caregivers from Canada and the Netherlands, respectively (Table 3).

### Health-related quality of life of caregivers and children with JIA

Table 2 presents the distribution of HRQoL responses (*n* = 250) on the five items of EQ-5D-5L. For their children with JIA, a higher proportion of responses reported severe or extreme problems in the domains ‘pain/discomfort’ (18.8%, *n* = 47) and ‘usual activities’ (14.0%, *n* = 35) compared to the other domains. Conversely, the highest percentage of ‘no problems’ was reported in the domain ‘self-care’ (67.6%, *n* = 169).

**Table 2:** The caregiver and child HRQoL (*n =* 250) on the five items of the EuroQol 5D-5L

Participants	Domains EQ-5D-5L				
	Mobility, n (%)	Self-care, n (%)	Usual activities, n (%)	Pain/discomfort, n (%)	Anxiety/depression, n (%)
Child (proxy-reported EQ-5D-5L-Y) (*n =* 250)					
No problem	111 (44.4)	169 (67.6)	89 (35.6)	57 (22.8)	103 (41.2)
Slight problem	58 (23.2)	41 (16.4)	82 (32.8)	76 (30.4)	105 (42.0)
Moderate problem	53 (21.2)	22 (8.8)	44 (17.6)	70 (28.0)	31 (12.4)
Severe problem	26 (10.4)	7 (2.8)	25 (10.0)	43 (17.2)	6 (2.4)
Unable to	2 (0.8)	11 (4.4)	10 (4.0)	4 (1.6)	5 (2.0)
Caregiver (self-reported EQ-5D-5L) (*n =* 250)					
No problem	197 (78.8)	236 (94.4)	195 (78.0)	141 (56.4)	180 (72.0)
Slight problem	36 (14.4)	8 (3.2)	28 (11.2)	70 (28.0)	49 (19.6)
Moderate problem	13 (5.2)	6 (2.4)	21 (8.4)	33 (13.2)	18 (7.2)
Severe problem	4 (1.6)	0	5 (2.0)	4 (1.6)	3 (1.2)
Unable to	0	0	1 (0.4)	2 (0.8)	0

*EQ-5D-5L* 5-level version of EuroQoL questionnaire, *EQ-5D-5L-Y* Youth 5-level version of EuroQoL questionnaire

For caregivers’ own health (*n* = 250), the proportion who reported severe, or extreme problems was < 3% for all domains, with the highest proportion observed in the ‘pain/discomfort’ and ‘usual activities’ domains (each 2.4%, *n* = 6). Most caregivers reported having ‘no problem’ with self-care (94.4%, *n* = 236), mobility (78.8%, *n* = 197), usual activities (78.0%, *n* = 195), and anxiety/depression (72.0%, *n* = 180). Table 3 presents the mean EQ-5D-5L utility score of 0.86 (SD 0.11, IQR 0.83–0.95) for Canadian caregivers’ own health (*n* = 72), and 0.89 (SD 0.16, IQR 0.85–1.00) for Dutch caregivers (*n* = 178). Approximately 40% of caregivers had ‘no problem’ in all of the EQ-5D-5L domains resulted in a health utility score equals to 1, generating a ceiling effect.

**Table 3:** Mean and median for caregiver EuroQol Five-Domain Questionnaire and CarerQoL utility scores and visual analog scale (*n =* 250) stratified by country of origin

	EQ-5D-5L		CarerQoL	
	Mean (SD)	Median (IQR)	Mean (SD)	Median (IQR)
**Utility score**				
Overall (*n =* 250)^a^	0.87 (0.16)	0.88 (0.82–1.00)	82.7 (11.4)	85.7 (79–90)
Canada (*n =* 72)^b^	0.86 (0.11)	0.90 (0.83–0.95)	80.1 (13.0)	81.9 (74–88)
Netherlands (*n =* 178) ^a^	0.89 (0.16)	0.89 (0.85–1.00)	83.7 (10.6)	87.1 (81–92)
**VAS score**				
Overall (*n =* 250)	﻿79.7 (17.1)	83.0 (58–90)	7.5 (1.5)	7.6 (7.0–8.5)
Canada (*n =* 72)	78.7 (19.5)	85.5 (70–91)	7.3 (2.1)	8.0 (6.5–9.0)
Netherlands (*n =* 178)	80.2 (16.1)	81.0 (75–90)	7.6 (1.16)	7.6 (7.0–8.3)

*EQ-5D-5L* 5-level version of EuroQoL questionnaire, *CarerQoL* Care-related quality of life questionnaire, *SD* Standard deviation, *IQR* Interquartile range, *VAS* Visual analogue scale

^a^EQ-5D-5L utility scores were calculated using value sets from the Netherlands; ^b^EQ-5D-5L utility scores were calculated using value sets from Canada

### Association between caregiver CRQoL and HRQoL and (proxy-reported) child HRQoL

Table 4 presents the Spearman’s correlation coefficients between children’s proxy-reported of EQ-5D-5L-Y domains and EQ-5D-5L-Y VAS, and the caregiver’s CarerQoL and EQ-5D-5L domains, as well as utility score and VAS. Lower caregiver’s CarerQoL utility scores were associated with more problems in anxiety/depression of their children. In addition, children’s problems with anxiety/depression were associated with statistical significance to more problems of caregivers with relational issues, daily activities, finances, and physical health.

**Table 4:** Spearman’s correlation coefficients of children’s proxy-reported EQ-5D-5L-Y domains and VAS, and caregiver CarerQoL and EQ-5D-5L domains, as well as utility scores and VAS

	Children’s EQ-5D-5L-Y domains (proxy-reported)					
	Mobility	Self-care	Usual activities	Pain/ discomfort	Worried/sad/unhappy	VAS
Caregiver’s CarerQoL domains						
Fulfilment	−0.11	−0.02	−0.12	− 0.08	− 0.09	0.14
Relational problems	0.16	0.11	0.19	0.15	0.36***	−0.15
Mental health problems	0.07	0.10	0.14	0.05	0.20	−0.17
Problems combining care tasks with daily activities	0.26**	0.28**	0.33**	0.19	0.30***	−0.23*
Financial problems	0.08	0.17	0.16	0.12	0.30***	−0.16
Support	−0.02	0.12	−0.03	− 0.03	− 0.04	0.12
Physical health problems	0.01	−0.01	0.11	0.05	0.22*	−0.08
Utility score	−0.15	−0.12	− 0.24	−0.14	− 0.32**	0.20
VAS	−0.11	−0.09	− 0.17	−0.14	− 0.20	0.26**
Caregiver’s EQ-5D-5L domains						
Mobility	0.20	0.03	0.21	0.24**	0.19	−0.20
Self-care	0.11	0.16	0.13	0.17	0.22*	−0.10
Usual activities	0.17	0.16	0.21	0.22*	0.23*	−0.26**
Pain/discomfort	0.13	−0.02	0.14	0.16	0.16	−0.20
Anxiety/depression	0.11	0.17	0.17	0.19	0.32***	−0.20
Utility score	−0.19	− 0.12	−0.23*	− 0.24*	−0.28***	0.25**
VAS	−0.13	−0.00	− 0.15	−0.17	− 0.14	0.5***

* *p* < 0.05;** *p* < 0.01; *** *p* < 0.001 using Bonferroni approach

Coefficient strength: perfect (1), very strong (0.8–0.99), moderate (0.6–0.79), fair (0.3–0.59), poor (0.1–0.29), and none (0–0.09)

*EQ-5D-5L* 5-level version of EuroQoL questionnaire, *EQ-5D-5L-Y* Youth 5-level version of EuroQoL questionnaire, *CarerQoL* Care-related quality of life questionnaire, *VAS* Visual analogue scale

There was statistically significant negative association with poor strength between caregivers’ EQ-5D-5L utility scores and problems for all domains with exception of mobility and self-care of children. In addition, increase in children’s problems with anxiety/depression were positively associated with increase in anxiety/depression of caregivers with fair strength.

### Factors associated with caregiver CRQoL and HRQOL utility scores

The multivariate regression analysis identified that higher CarerQoL utility scores were associated with living in the Netherlands, being employed, and higher EQ-5D-L-Y utility scores (Table 5). Lower CarerQoL utility scores were associated with receiving paid or unpaid help.

**Table 5:** Results of multilinear regression analysis to identify factors associated with caregiver’s CarerQoL and EQ-5D-5L utility scores, respectively

	CarerQoL		EQ-5D-5L (two-part model)			
	OLS regression with robust standard errors results		Logistic regression model for probability of reaching full health score (EQ-5D-5L = 1)		OLS regression results for caregivers with EQ-5D-5L utility scores less than 1	
Variables, reference	Coefficient (SE)	*P* value	Coefficient (SE)	*P* value	Coefficient (SE)	*P* value
Constant	61.45 (7.22)	< 0.01	0.63 (1.41)	0.65	0.53 (0.13)	< 0.01
Child’s age (years)	−0.15 (0.20)	0.47	−0.06 (0.04)	0.15	−0.01 (0.00)	0.02
Child’s gender, female	1.11 (1.42)	0.44	−0.47 (0.31)	0.13	0.00 (0.02)	0.94
Disease status, active	2.54 (1.98)	0.20	−0.14 (0.41)	0.73	−0.05 (0.03)	0.18
Subcutaneous therapy, yes	−1.54 (1.69)	0.36	0.49 (0.35)	0.16	0.04 (0.03)	0.20
EQ-5D-5L-Y utility score	10.51 (3.57)	< 0.01	1.52 (0.63)	0.02	0.15 (0.03)	< 0.01
Caregiver’s age (years)	−0.03 (0.11)	0.75	− 0.03 (0.03)	0.23	0.00 (0.00)	0.36
Caregiver’s gender, female	0.63 (1.94)	0.74	−0.87 (0.40)	0.03	0.02 (0.04)	0.51
Country, Netherlands	4.99 (1.71)	< 0.01	0.41 (0.34)	0.23	0.03 (0.03)	0.23
Employment status, employed	7.32 (2.27)	< 0.01	1.00 (0.41)	0.01	0.04 (0.03)	0.14
Receive paid or unpaid help, yes	−6.72 (2.09)	< 0.01	−0.43 (0.42)	0.31	−0.08 (0.03)	0.02
Living with spouse, yes	6.74 (3.65)	0.06	0.09 (0.55)	0.86	0.05 (0.04)	0.23
Observations	217		217		114	
R^2^	0.25		–		0.16	

*EQ-5D-5L* 5-level version of EuroQoL questionnaire, *EQ-5D-5L-Y* Youth 5-level version of EuroQoL questionnaire, *CarerQoL* Care-related quality of life questionnaire, *SE* Standard error, *OLS* Ordinary least square

For caregiver HRQoL, caregivers who were employed, male gender, or whose children had a higher EQ-5D-5L-Y utility score were more likely to have an optimal health utility score (EQ-5D-5L utility equals 1). Among caregivers who scored less than 1 in EQ-5D-5L, higher EQ-5D-5L-Y utility score were positively associated with caregiver’s EQ-5D-5L utility score (*p* < 0.01). In addition, child’s age and receiving paid or unpaid help were negatively associated with caregiver’s EQ-5D-5L utility score (*p* < 0.05).

Neither disease activity status nor administration of subcutaneous therapy were associated with statistical significance to caregiver’s CarerQoL or EQ-5D-5L utility scores.

## Discussion

Caregiver care-related and health-related quality of life are crucial to a broader understanding of the JIA burden beyond its effects on the patient alone. In this study, we described the results of caregiver CRQoL and HRQoL, as measured by CarerQoL and EQ-5D-5L, for a sample of 250 caregivers from Canada and the Netherlands. This is the first study to evaluate CRQoL using CarerQoL questionnaire in caregivers of children with JIA and to explore the potential relationship of caregiver HRQoL and CRQoL with child and caregiver’s characteristics, and other caring context variables.

In this study, we observed a higher number of participants from the Netherlands (*n* = 178/250) than from Canada (*n* = 78/250). This difference had two major contributors: the recruitment of patients and caregivers started earlier in the Netherlands than Canada, and recruitment through the year of 2021 was halted in Canada, but not in the Netherlands, due to the pandemic. Although differences number of respondents between countries, patients were consecutively invited to participate of the UCAN CANDU study and the characteristics between respondents and non-respondents were similar, pointing to a low risk of selection bias.

Our assessment of child HRQoL using proxy-reported EQ-5D-5L-Y point to ‘pain/discomfort’ and ‘usual activities’ as the most affected domains of children’s health. A recent study reporting the responses for EQ-5D-5L-Y for 68 patients with JIA also found a higher proportion of problems in these two domains [38]. However, we observed in our cohort a higher proportion of children with severe and extreme problems in all domains of the EQ-5D-5L-Y. This difference could be because our cohort had a higher proportion of patients with active disease status in our cohort (75% compared with 43%). The level of disease activity in our study reflects a selected cohort of patients enrolled in the UCAN CADU study who are either getting a JIA diagnosis, starting biologics, or stopping biologics.

The assessment of caregiver HRQoL using the EQ-5D-5L questionnaire revealed a high mean utility score, with almost 40% of parents presenting full health (utility equals to 1). The mean EQ-5D-5L utility score reported in our study (0.86 and 0.89 for Canadian and Dutch caregivers, respectively) was comparable to age-specific population norms reported in Canada and the Netherlands (0.85 and 0.85, respectively) [34, 39]. Conversely, the mean EQ-5D-5L utility scores we observed were substantially higher than those reported by the only other study reporting mean utility scores of caregivers of children with JIA (between 0.38 to 0.80 depending on the country) [17]. This difference could be due to the latter study’s very limited sample size (between 1 and 16 respondents per country).

This is the first study reporting on CRQoL using CarerQoL utility scores in caregivers of children with JIA, therefore we are only able to compare our findings with studies focused on other childhood conditions. The mean CarerQoL utility scores reported in our cohort (mean: 83) is comparable with the mean scores reported for mothers of children with cystic fibrosis (mean: 84, *n* = 130) and caregivers of children with drug-resistant epilepsy (mean: 81, *n* = 181, 25, 26]. However, the scores we found were higher than in a study reporting CarerQoL utility score for caregivers of children with an autism spectrum disorder (mean: 77, *n* = 76), which reports a higher proportion of relational problems with the care receiver [40].

We assessed the potential association between caregiver CRQoL and HRQoL domains and child HRQoL domains. Our results show that higher levels of children’s ‘pain/discomfort’ were associated with two caregiver HRQoL domains (i.e., mobility and usual activities) and utility scores. This finding is supported by studies that indicated pain management is an especially challenging aspect of JIA and impacts parent’s usual activities [19]. In addition, our analysis showed that children’s feelings of ‘sadness/unhappiness’ is associated with caregiver's anxiety/depression. This finding was consistent with a literature review showing that poorer parental mental health (i.e., depression, depressive symptoms, or anxiety) was associated with greater prevalence of depression or depressive symptoms in the child [41]. Finally, we showed children’s increased levels of sadness and/or unhappiness play a major role in parent CRQoL and are associated with increasing problems in all negative aspects of caregiving in CarerQoL. While we did not identify other studies that directly evaluate the effects of children’s sadness/unhappiness, this finding is consistent with literature highlighting the substantial impact of children’s depressive symptoms on families [41].

Beyond the association of specific domains between child HRQoL and caregiver HRQoL and CRQoL, one of the main findings of this study is that child EQ-5D-5L-Y utility scores had a positive relationship with both caregiver’s CarerQoL and EQ-5D-5L utility scores. These findings are supported by a study indicating a pooled moderate to strong relationship between parent and child well-being, although these findings were not specific to health utility scores [19]. As this is the first time the relationship between utility scores was assessed in JIA, there are no studies to which to compare the magnitude of this result. However, in a study examining the relationship between EQ-5D-5L utility scores of caregivers and children with meningitis, Al-Janabi and colleagues found an identical coefficient (0.16) in their multivariate analysis [42]. Additionally, in another study focused on caregivers of patients suffering from multiple diseases, caregiver’s CarerQoL and EQ-5D-5L utility scores were found to be associated to the care recipient EQ-5D-5L health status (correlation coefficient of 0.30 and 0.24, respectively) [22].

Interestingly, despite child HRQoL having a substantial impact on CarerQoL and EQ-5D-5L utility scores, we found that disease activity status was not associated with either score. Other studies have shown that JIA disease activity is not always aligned with the intensity of children’s pain, fatigue, or overall quality of life [43, 44]. These findings would explain our results since child’s pain and well-being are two factors that are prioritized by parents as shown in qualitative evidence, which would be part of the HRQoL measurement in this study [12, 19].

Having a job is associated with higher caregiver’s CarerQoL and EQ-5D-5L utility scores, a result consistent with another study [22]. This finding may indicate parents with perfect EQ-5D-5L scores or higher CarerQoL scores are more likely able to balance employment with their child’s care or, alternatively, parents who are able to balance employment with their child’s care are able to maintain their jobs. Also, parents receiving paid or unpaid help was associated with lower CarerQoL and EQ-5D-5L utility scores. We hypothesize that caregivers with higher care burden, captured by lower CRQoL or HRQoL are more likely to need either paid or unpaid help to support caring for their child and/or household chores. Living in the Netherlands was also identified as positively associated with CarerQoL scores. This finding is aligned with results from the latest United nation Children’s Fund report, which ranked Netherlands higher than Canada in the dimensions evaluating child’s well-being, family, education and health policies, and economic and social context including whether parents have the support and resources to give their children the best chance for a healthy, happy childhood [45].

The caring context factors associated with caregiver CRQoL and HRQoL highlight the need for an encompassing family-centred approach of care that goes beyond achieving inactive disease. If programs and services target only families with children experiencing active disease status, families with children that do not have active disease will not be adequately supported, although they might have significant caregiving burden. By assessing caregiver burden, caregivers at risk can be identified, which enables health professionals and policy makers to actively offer programs and services to support families at an early stage. This may include external care provision, employment counselling, or financials aids.

One of the limitations of this study is that scoring algorithms are not yet available for the 5-level EQ-5D-Y instrument. Although research suggests that adult value sets are not suitable to be used to calculate EQ-5D-Y utility scores [46], in this study, we used adult value sets as a placeholder while research advances in this field, assuming that final value sets are not too different from this proxy. We also used Dutch CarerQoL-7D value sets as Canadian value sets are not available yet. The impact of having used value sets from the Netherlands is unknown as we cannot predict how Canadians would value CarerQoL health states. However, for the regression analysis, given differences in values among health states remains similar, we would not expect changes in our findings. Finally, the variables included in the HRQoL model explained 16% of EQ-5D-5L utility score variability. Therefore, further studies are needed to investigate other factors such as duration of the disease, as well as investigating these relations in more flexible models, including non-linear models. Moreover, potentially complex relationships between variables may warrant analysis of longitudinal data.

## Conclusion

We conclude that HRQoL of children with JIA is associated with their caregiver CRQoL and HRQoL. In addition, to understand the impact of JIA on families, we need to consider not only children’s disease activity status, but also socio-economic factors such as employment and support to carry care-giving tasks. The findings presented in this study highlight the need to further investigate the factors associated with caregiver CRQoL and HRQoL. Furthermore, there is a need for research on the impact of practical application of the CRQoL utility scores on economic evaluation studies.

## Data Availability

The data that support the findings of this study are available from UCAN CAN-DU and UCAN CURE consortia, but restrictions apply to the availability of these data, which were used under license for the current study, and so are not publicly available. Data are however available from the authors upon reasonable request and with permission of UCAN CAN-DU and UCAN CURE consortia.
